# Transcriptome analysis reveals seasonal dynamics and oil yield associated regulatory networks in *Camphora longepaniculata*

**DOI:** 10.3389/fpls.2026.1875536

**Published:** 2026-07-08

**Authors:** Zhou Xu, Tianfei Dai, Zhonghua Liang, Qi Zhong, Mengyang Du, Junqiang Li, Xin Liu, Lina Meng, Wenwen Liu, Li Yang, Jian Zhang, Mengling Wen, Kuan Yan

**Affiliations:** 1 Faculty of Agriculture, Forestry and Food Engineering, Yibin University, Yibin, China; 2 Sichuan Oil Cinnamon Engineering Technology Research Center, Yibin University, Yibin, China; 3 Sichuan Green Food Development Center, Chengdu, China; 4 Xingwen Sanhe Landscaping Co., Ltd, Yibin, China; 5 Rongxian Agricultural Development Guarantee Center, Rongxian, China

**Keywords:** *Camphora longepaniculata*, oil-yield variation, seasonal transcriptome, terpenoid biosynthesis, WGCNA

## Abstract

*Camphora longepaniculata* is a terpene-rich aromatic tree valued for its cineole-dominant essential oils, but the molecular basis of its seasonal dynamics and variation in oil yield remains unclear. Here, we profiled leaf transcriptomes from high, medium, and low oil lines collected across four seasons and used a multifactor framework to separate the effects of season, oil type, and their interaction on gene expression. High-quality RNA-seq data were obtained from 34 libraries. Global transcriptome analyses showed that season was the primary source of expression variation, whereas oil-yield class contributed a stable but secondary component to transcriptional divergence. Differential expression analysis indicated that differences among oil-yield types were most pronounced in autumn and winter, particularly between the low-oil line and the high- and medium-oil lines, while seasonal transcriptional changes varied among chemotypes. Main-effect analysis identified large sets of genes associated with season and oil type. Clustering of season-responsive genes resolved seven temporal expression modules, including winter-induced, autumn-biased, summer-activated, and spring-preferential patterns, indicating extensive transcriptome reprogramming over the annual cycle. In parallel, oil-type-responsive genes clearly distinguished the low-oil materials from high- and medium-oil materials, supporting a stable transcriptional program linked to oil accumulation. Weighted gene co-expression network analysis identified a trait-associated module that was positively correlated with high oil content and enriched for signaling, membrane-associated, and regulatory functions. Hub candidate genes in this module included ABC transporters, O-methyltransferases, cytochrome P450s, UDP-glycosyltransferases, and a terpene synthase. Consistent with these patterns, multiple genes in the MVA and MEP pathways showed higher expression in the high-oil line, suggesting a possible association with terpene precursor supply. Together, these results indicate that essential-oil accumulation in *C. longepaniculata* is associated with a stable oil-type-related transcriptional program overlaid with strong seasonal reprogramming, and they provide candidate genes and regulatory modules for future functional studies and molecular breeding of high-oil germplasm.

## Introduction

*Camphora longepaniculata* (Gamble) Y. Yang, Bing Liu, and Zhi Yang is an evergreen aromatic tree in the Lauraceae that is widely recognized for its high essential-oil content and economic importance in southwestern China (Huang et al., 2025). Its leaves and branchlets are widely used as raw materials for extracting volatile oils for pharmaceutical, fragrance, and daily chemical applications. These oils, which are rich in terpenoids, contain 1,8-cineole, α-terpineol, and γ-terpinene as the main components (Li et al., 2014; Yan et al., 2017). Beyond their industrial value, these compounds exhibit antibacterial, antioxidant, and anti-inflammatory activities, highlighting the medicinal relevance of *C. longepaniculata* as a terpene-producing woody species (Chen et al., 2018). Recent studies have also shown that oil yield and composition are influenced by extraction methods, developmental stage, environmental conditions, and varietal background, indicating that both genetic and environmental factors contribute to essential-oil accumulation (Zhao et al., 2023).

Terpenoids are the largest and one of the most diverse classes of plant secondary metabolites and play key roles in defense, signaling, ecological interactions, and environmental adaptation (Bohlmann and Keeling, 2008). To provide a clearer overview of terpene diversity, representative terpene compounds, their major terpene classes, and corresponding precursor pathways are summarized in **Table 1**. Their biosynthesis relies on the coordinated activity of the mevalonate (MVA) and methylerythritol phosphate (MEP) pathways, followed by diversification of intermediates by terpene synthases (TPSs), which are central to terpene structural diversity (McGarvey and Croteau, 1995; O’maille et al., 2008). In *C. longepaniculatum*, previous studies have begun to clarify the molecular basis of terpene production. Early transcriptome analyses identified genes involved in monoterpene biosynthesis (Yan et al., 2017), and later work showed that endophytic fungi can alter the expression of essential-oil biosynthetic genes and affect oil accumulation (Yan et al., 2019). More recent integrative analyses combining transcriptomics and metabolomics across varieties have linked variation in oil content and leaf traits to changes in secondary metabolism, including terpenoid pathways (Zhao et al., 2022, Zhao et al., 2023). Together, these findings suggest that terpene accumulation in *C. longepaniculata* is controlled by a coordinated regulatory network responsive to both developmental and environmental cues.

**Table 1:** Representative terpene compounds, terpene classes, major biosynthetic pathways, and references.

Terpene class	Representative compounds	Biological or industrial relevance	References
Monoterpenes	1,8-cineole; α-terpineol; γ-terpinene; linalool; limonene	Mainly MEP pathway; GPP as direct precursor	McGarvey and Croteau, 1995; Dudareva et al., 2013; Tholl, 2006; Yan et al., 2017; Li et al., 2014
Sesquiterpenes	β-caryophyllene; farnesene; nerolidol; artemisinin-related sesquiterpenes	Mainly MVA pathway; FPP as direct precursor	McGarvey and Croteau, 1995; Tholl, 2006; Dudareva et al., 2013; Nagegowda, 2010
Diterpenes	Gibberellins; phytol; taxadiene; labdane-type diterpenes	Mainly MEP pathway; GGPP as direct precursor	McGarvey and Croteau, 1995; Vranová et al., 2013; Nagegowda, 2010
Triterpenes	Squalene; sterols; β-amyrin; oleanolic acid	Mainly MVA pathway; squalene derived from FPP	McGarvey and Croteau, 1995; Vranová et al., 2013
Tetraterpenes	Carotenoids; β-carotene; lutein; zeaxanthin	Mainly MEP pathway; GPP as direct precursor	McGarvey and Croteau, 1995; Vranová et al., 2013

The availability of genomic resourced has enables more detailed investigation of this network. A chromosome-level genome assembly of *C. longepaniculata* provides a high-quality reference for gene identification, comparative genomics, and pathway analysis (Yan et al., 2024). Building on this resource, a genome-wide survey of the TPS family identified 86 TPS genes, revealed lineage-specific expansion within Lauraceae, and showed that some TPS genes are preferentially upregulated in high-oil materials (Liu et al., 2025). While these studies have advanced our understanding of the genomic basis of terpenoid biosynthesis, they do not fully explain how transcriptional programs vary across seasons or why different oil-content types maintain distinct metabolic states under changing environmental conditions. This question is particularly relevant because the physiology and distribution of *C. longepaniculata* are strongly influenced by climate, and recent ecological analyses indicate sensitivity to temperature and precipitation across its range (Zhu et al., 2025). Understanding how seasonal variation interacts with intrinsic oil-content differences therefore remains important for both biological insight and practical applications.

Despite growing interest in the phytochemistry, pharmacology, genomics, and TPS evolution of *C. longepaniculatum*, a systematic transcriptomic framework that simultaneously evaluates seasonal effects, oil-type effects, and their interaction is still lacking. Such an approach is necessary because seasonal transcriptional changes may obscure or modify constitutive differences among high-, medium-, and low-oil materials, whereas genes that remain consistently associated with oil type across seasons are more likely to represent core regulators of essential-oil accumulation. In this study, we conducted a multifactor transcriptome analysis across four seasons and three oil-content types in *C. longepaniculata*. By integrating global expression profiling, multifactor differential expression analysis, interaction analysis, and expression pattern clustering, we aimed to distinguish genes primarily driven by seasonal variation from those associated with stable oil-type differences, and to identify candidate regulators influenced by both factors. This work provides a transcriptome-level framework for understanding seasonal regulation of oil accumulation in *C. longepaniculata* and offers candidate genes and molecular modules for future functional studies and germplasm improvement.

## Materials and methods

### Plant material, sampling, and RNA extraction

Leaf samples of *Camphora longepaniculata* were collected from Sichuan Province, China (27°50′ N, 105°20′ E). Three *C. longepaniculata* lines with different leaf oil contents were used in this study, including line 5# with a high oil content (0.06 mg/g DW), line 7# with a medium oil content (0.053 mg/g DW), and line 14# with a low oil content (0.047 mg/g DW). The oil contents of these three lines were measured in December and expressed as milligrams of extracted oil per gram of leaf dry weight (mg/g DW). These December oil-content values were used to classify the materials into high-, medium-, and low-oil groups for transcriptome analysis. To investigate seasonal transcriptomic variation among different oil-yielding lines, healthy leaves with similar growth status were collected from the three lines in March, June, September, and December. For each line at each seasonal sampling time point, three biological replicates were collected from comparable positions on the same individual plant to minimize developmental and positional variation. All collected samples were immediately frozen in liquid nitrogen and stored at −80 °C until RNA extraction.

Total RNA was extracted from frozen leaf tissues using the RNAprep Pure Plant Kit (Tiangen Biotech Co., Ltd., Beijing, China) according to the manufacturer’s instructions. RNA concentration and purity were measured using a NanoDrop 2000 spectrophotometer (Thermo Fisher Scientific, Wilmington, DE, USA). RNA integrity was evaluated using the RNA Nano 6000 Assay Kit on an Agilent Bioanalyzer 2100 system (Agilent Technologies, CA, USA). Only RNA samples with adequate concentration, purity, and integrity were used for subsequent cDNA library construction and transcriptome sequencing.

### Transcriptome sequencing and data analysis

The reference genome sequence and corresponding GFF annotation file of *Camphora longepaniculata* were downloaded from Figshare (https://figshare.com/articles/dataset/_b_i_Camphora_longepaniculata_i_b_genome/24564532) (Yan et al., 2024). The transcriptome sequencing data used in this study have been deposited in the NCBI Sequence Read Archive (SRA) database under accession number PRJNA1458560. The raw sequencing data generated in FASTQ format were first subjected to quality control. Adapter sequences, reads with a high proportion of ambiguous bases, and low-quality reads were removed using custom Perl scripts. The remaining high-quality clean reads were used for all subsequent analyses. Clean reads from each library were aligned to the *C. longepaniculata* reference genome using HISAT2 with default parameters suitable for spliced alignment (Kim et al., 2015). The mapping results were examined to evaluate sequencing quality and alignment efficiency among samples. The mapped reads were then assembled and quantified using StringTie based on the reference genome annotation (Pertea et al., 2015). Gene expression levels were calculated from the read alignment results and used for downstream comparative transcriptomic analyses.

Differential expression analysis was performed using the DESeq2 package in R (Love et al., 2014). Raw read counts were used as input for DESeq2, and sequencing depth and RNA composition differences among libraries were normalized using the median-of-ratios method implemented in DESeq2. The normalized expression matrix was used for exploratory analyses, including sample-to-sample Pearson correlation analysis, principal component analysis (PCA), and hierarchical clustering, whereas raw count data were retained for formal statistical testing in DESeq2. Pairwise comparisons were conducted among different oil-yielding lines within each sampling season and among different sampling seasons within each oil-yielding line. P values were adjusted for multiple testing using the Benjamini–Hochberg false discovery rate method. Genes with an adjusted *P* value < 0.05 and an absolute log_2_ fold change value ≥ 1 were identified as differentially expressed genes (DEGs). The resulting DEG sets were used for subsequent clustering, functional enrichment, and pathway-related analyses.

### Multifactor differential expression analysis

Differential expression analysis was performed using the DESeq2 package in R based on raw read count data. Before statistical testing, raw counts were normalized using the DESeq2 median-of-ratios method. Pairwise comparisons were conducted among oil-yield types within each season and among seasons within each oil-yield type. P values were adjusted for multiple testing using the Benjamini–Hochberg false discovery rate correction. Genes with an adjusted *P* value < 0.05 and an absolute log2FoldChange ≥ 1 were defined as differentially expressed genes. To evaluate the effects of season, oil type, and their interaction on gene expression, differential expression analysis was performed in DESeq2 using a multifactor design. Raw count data were used for the formal analysis, with the design formula specified as Season + OilType + Season: OilType. Season included spring, summer, autumn, and winter, and OilType included high-, medium-, and low-oil groups. Likelihood ratio tests (LRTs) were used to identify genes significantly associated with the main effects of Season and OilType, as well as the Season × OilType interaction. Genes with Benjamini–Hochberg adjusted *P* values < 0.05 were considered significant in the multifactor analysis. Wald tests were subsequently used for pairwise contrasts to determine the direction and magnitude of expression changes between specific seasons or oil-yield groups.

### Pattern analysis and visualization of responsive genes

Season-responsive genes were further analyzed to characterize dynamic expression patterns across spring, summer, autumn, and winter. Average expression values were calculated for each season, standardized, and subjected to fuzzy c-means clustering using the Mfuzz package (Kumar and Futschik, 2007) to identify representative seasonal expression modules. For OilType-responsive genes, average expression profiles were calculated for the high-, medium-, and low-oil groups and visualized as heatmaps to reveal stable transcriptional differences associated with oil accumulation. To assess whether oil-type effects were maintained across seasonal backgrounds, expression patterns of representative genes were also summarized across all 12 Season × OilType combinations. Genes with significant Season × OilType interaction effects were regarded as candidates involved in oil-type-specific seasonal regulation. All statistical analyses and visualizations were conducted in R using DESeq2, ggplot2, pheatmap, ComplexHeatmap, dendextend, and Mfuzz.

### Weighted gene co-expression network analysis

To identify gene modules associated with seasonal variation and oil-content type, we performed weighted gene co-expression network analysis (WGCNA) in R using the WGCNA package (Langfelder and Horvath, 2008). Genes with low expression levels or low variance across samples were excluded before network construction to reduce noise. Pairwise Pearson correlation coefficients were calculated among the retained genes, and an adjacency matrix was constructed using a soft-thresholding power chosen based on the scale-free topology criterion. Based on the scale-independence and mean-connectivity analyses, the soft-thresholding power was set to β = 9. At this value, the scale-free topology fit index reached R² = 0.79, which was close to the commonly used threshold of 0.80, while the mean connectivity remained sufficient for module detection. In addition, the log10(k)–log10(p(k)) relationship showed an approximately linear distribution at β = 9, indicating that the constructed network satisfied the scale-free topology assumption. This matrix was then transformed into a topological overlap matrix (TOM), and genes were hierarchically clustered according to TOM-based dissimilarity. Co-expression modules were identified using the dynamic tree cut algorithm with a defined minimum module size, and modules with highly similar expression profiles were merged based on module eigengene correlations. Module eigengenes were then correlated with external traits to identify biologically relevant modules. Genes within trait-associated modules were treated as candidate genes for further analysis, and hub genes were prioritized based on high module membership and strong gene–trait correlations.

### Quantitative real-time PCR analysis

The relative transcript levels of selected genes were examined by quantitative real-time PCR (qRT-PCR). First-strand cDNA was synthesized from total RNA and used as the template for amplification. Actin was used as the internal reference gene for normalization. qRT-PCR was performed on a CFX96 Real-Time PCR Detection System using TB Green Premix Ex Taq II (Tli RNaseH Plus). Each 10 μl reaction contained 5 μl of 2× premix, 0.4 μl of each forward and reverse primer (10 μM), 1 μl of diluted cDNA template, and nuclease-free water to the final volume. The thermal cycling program consisted of an initial denaturation step at 95 °C for 3 min, followed by 39 cycles of 95 °C for 10 s and 58 °C for 30 s. Amplification specificity was confirmed by melting-curve analysis, performed after PCR by increasing the temperature from 65 °C to 95 °C at a rate of 0.5 °C per minute following a brief denaturation step at 95 °C for 5 s. Gene-specific primers were designed using Primer Premier 5.0, and detailed primer information is provided in **Supplementary Table 1**. Relative expression levels were calculated after normalization to Actin, and statistical differences among groups were assessed by one-way ANOVA followed by multiple-comparison tests. Expression data were visualized using GraphPad Prism 7.04, with significance levels indicated as *, **, and ***.

## Results

### RNA-seq quality assessment

To characterize seasonal transcriptomic variation among *Camphora longepaniculata* chemotypes with contrasting leaf oil yields, RNA-seq libraries were constructed from high, medium and low oil yielding lines at four seasonal sampling points, with biological replicates included for each group (**Supplementary Table 2**). After quality filtering, the 34 libraries generated 39.15 to 59.04 million clean reads per sample, corresponding to 5.87 to 8.86 Gb of clean data. The sequencing data showed high quality, with error rates of 0.02 to 0.03%, Q20 values of 97.27 to 98.21% and Q30 values of 92.75 to 94.92%. GC content was highly consistent across libraries, ranging from 45.59 to 47.51%, which indicated no obvious GC bias. Clean reads mapped to the reference genome at rates of 72.69 to 91.69%, with an overall mapping rate of 85.49%. The summer libraries showed the highest average mapping efficiency, reaching 90.54%, whereas quality metrics remained stable across oil yield classes and seasons. Together, these results indicate that the RNA-seq datasets were robust, reproducible and suitable for subsequent comparative transcriptomic analyses of seasonal regulation and oil yield associated gene expression.

### Sample correlation, PCA and hierarchical clustering

**Figure 1:**
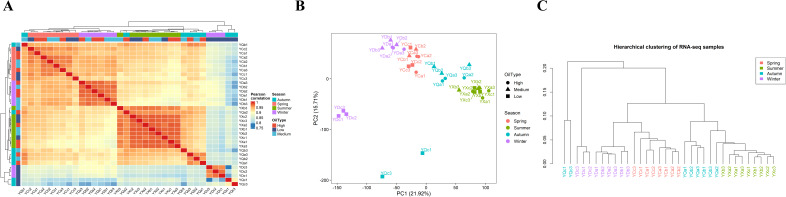
Global transcriptome relationships among *Camphora longepaniculata* samples across seasons and oil-yield types. **(A)** Pearson correlation heatmap based on normalized RNA-seq expression profiles. The color scale indicates the Pearson correlation coefficient between samples. **(B)** PCA of all RNA-seq libraries. Point colors indicate season, and point shapes indicate oil-yield type. **(C)** Hierarchical clustering based on normalized global expression profiles.

To assess the consistency of biological replicates and the global structure of the RNA-seq datasets, pairwise correlation analysis, principal component analysis and hierarchical clustering were performed using normalized expression profiles. The sample correlation matrix showed high overall reproducibility among libraries, with most pairwise Pearson correlation coefficients ranging from 0.75 to 1.00. Samples collected in the same season generally clustered together, indicating that seasonal variation was a major source of transcriptomic differentiation (**Figure 1A**). In the principal component analysis, the first two principal components explained 37.9% of the total variance, with PC1 and PC2 accounting for 21.9% and 16.0%, respectively. The samples were separated primarily by season. Summer samples formed a distinct cluster on the positive side of PC1, winter samples were mainly distributed on the negative side of PC1, and spring samples occupied an intermediate position (**Figure 1B**). Autumn samples were more dispersed, suggesting greater transcriptomic heterogeneity during this period.

Hierarchical clustering further supported this pattern, as samples were broadly grouped by seasonal origin rather than by oil yield type. Most biological replicates from the same seasonal and oil yield groups clustered closely, confirming the reliability of the RNA-seq data for downstream comparative analyses (**Figure 1C**). A small number of samples, including YQc1 and YQc3, showed relatively large separation from their corresponding groups in the principal component and clustering analyses, suggesting that these libraries may reflect biological variation or sample specific expression differences. Overall, these results indicate that the transcriptomic profiles were robust and that season was the dominant factor shaping global gene expression variation among oil yielding *Camphora longepaniculata* lines.

In addition, these exploratory analyses did not reveal an obvious technical batch effect. Samples were mainly separated by biological factors, especially season, rather than by sequencing-related artifacts. Therefore, the observed transcriptomic variation was considered to primarily reflect seasonal and oil-yield-associated biological differences.

### Differential gene expression analysis

**Figure 2:**
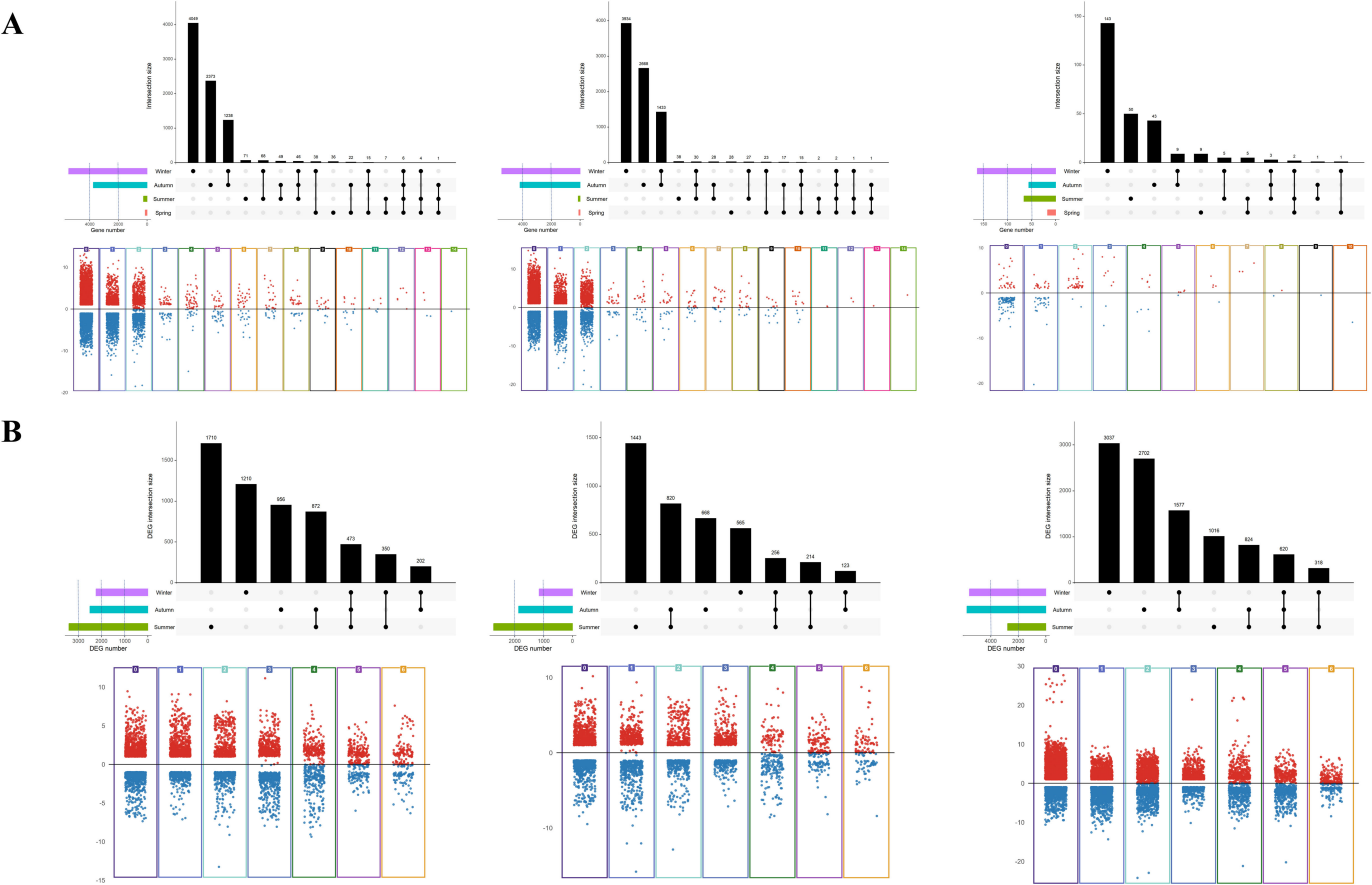
Differentially expressed genes among oil-yield types and across seasons in *Camphora longepaniculata*. **(A)** UpSet plots showing the overlap of DEGs across seasons for pairwise comparisons between oil-yield types. From left to right, the panels correspond to High vs Low, Medium vs Low, and High vs Medium. **(B)** UpSet plots of seasonal DEGs within high-, medium-, and low-oil lines, using spring as the reference. DEGs were identified using DESeq2 with Benjamini–Hochberg adjusted P value < 0.05 and |log_2_FoldChange≥ 1.

Differentially expressed genes were identified to characterize transcriptional differences among oil yield types within each season and seasonal transcriptional shifts within each oil yield type. In comparisons among oil yield types, the DEG landscape was strongly dependent on season. In the high versus low oil comparison, the largest season specific DEG set was detected in winter, with 4,049 DEGs, followed by autumn, with 2,373 DEGs. A total of 1,308 DEGs were shared between winter and autumn, whereas summer and spring specific differences were much smaller. A similar pattern was observed in the medium versus low oil comparison, in which winter specific DEGs were again the most abundant, followed by autumn specific DEGs and a substantial winter and autumn overlap. By contrast, the high versus medium oil comparison produced markedly fewer DEGs overall, with only 143 winter specific, 50 summer specific and 43 autumn specific DEGs (**Figure 2A**). This result indicates that the high and medium oil lines were transcriptionally more similar to each other than either was to the low oil line. The corresponding volcano plots further confirmed that the strongest transcriptional contrasts occurred in comparisons involving the low oil line, particularly in winter and autumn.

Seasonal DEG analysis within each oil yield type was then performed using spring as the reference. In the high oil line, summer showed the largest season specific transcriptional shift, with 1,710 summer specific DEGs, followed by winter and autumn specific DEG sets and several shared seasonal responses. The medium oil line showed a similar pattern, with the largest DEG set also specific to summer, followed by autumn and winter related responses. In contrast, the low oil line showed a distinct seasonal pattern, with winter and autumn specific DEGs being the most prominent, including 3,007 winter specific and 2,702 autumn specific DEGs, together with a large DEG set shared between winter and autumn (**Figure 2B**). These results indicate that transcriptional divergence among oil yield types was most pronounced under winter and autumn conditions, especially when the low oil line was compared with the high or medium oil lines. Seasonal response patterns within each material differed among oil yield types, with summer associated changes predominating in the high and medium oil lines and winter and autumn associated changes predominating in the low oil line.

### Seasonal main-effect genes define distinct transcriptional programs in *Camphora longepaniculata*

**Figure 3:**
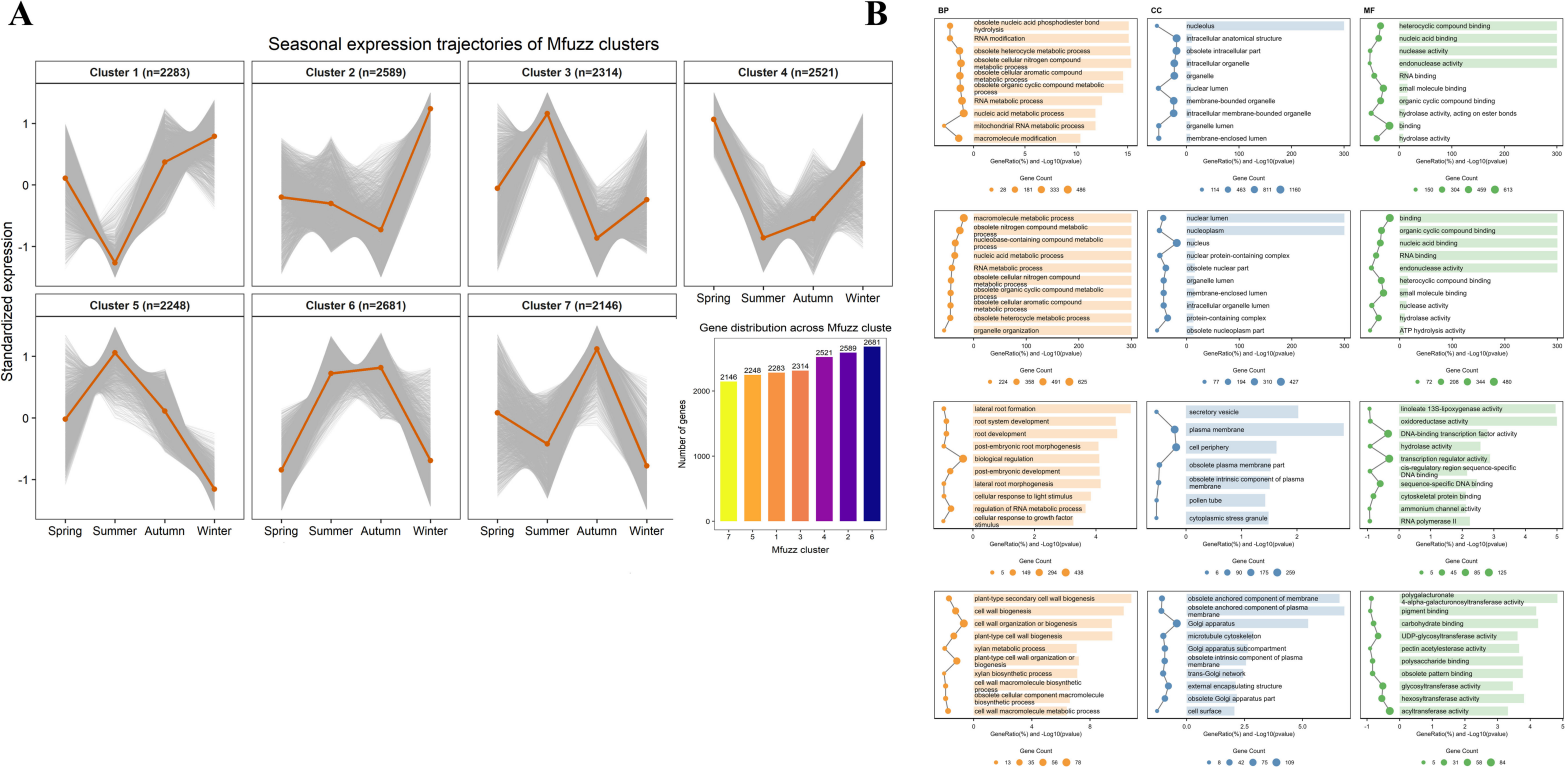
Seasonal expression clustering and GO enrichment analysis of season-responsive genes in *Camphora longepaniculata*. **(A)** Mfuzz clustering of genes with significant seasonal main effects. Gray lines indicate individual standardized expression profiles, orange lines indicate cluster means, and the inset shows gene numbers per cluster. **(B)** GO enrichment analysis of selected seasonal clusters. From top to bottom, the four rows correspond to Cluster 2, Cluster 3, Cluster 5, and Cluster 6. For each cluster, the left, middle, and right panels show enriched terms in the BP, CC, and MF categories, respectively. Bubble size reflects gene number, and bubble color indicates enrichment significance.

To identify transcriptional changes associated with season independently of oil-yield class, a multifactor DESeq2 analysis using a model including Season, OilType, and the Season × OilType interaction. The main effect of season was tested using a likelihood ratio test (LRT) while controlling for oil type and interaction-related variation. Genes with a significant seasonal effect were then analyzed by Mfuzz soft clustering, which identified seven major seasonal expression modules. These clusters were similar in size, ranging from 2,146 to 2,681 genes, including Cluster 1 (n = 2,283), Cluster 2 (n = 2,589), Cluster 3 (n = 2,314), Cluster 4 (n = 2,521), Cluster 5 (n = 2,248), Cluster 6 (n = 2,681), and Cluster 7 (n = 2,146) (**Figure 3A**). The relatively even distribution of genes across clusters suggests that the seasonal transcriptome is organized into several coordinated temporal programs rather than being dominated by a single expression pattern.

The seven clusters exhibited distinct seasonal trajectories. Cluster 1 was reduced in summer and then gradually recovered through autumn and winter (**Figure 3A**). Cluster 2 remained relatively stable from spring to summer, declined further in autumn, and was strongly induced in winter. Cluster 3 showed a transient summer peak, reaching maximal expression in summer, followed by a sharp decline in autumn and partial recovery in winter. Cluster 4 displayed the opposite pattern, with relatively high expression in spring, marked repression in summer and autumn, and moderate reactivation in winter. Cluster 5 reached its highest expression in summer and then declined gradually through autumn and winter. Cluster 6 was low in spring, elevated in summer and autumn, and sharply downregulated in winter. Cluster 7 showed a clear autumn-biased pattern, with low expression in summer, strong induction in autumn, and repression in winter. Together, these results indicate that season-responsive genes are partitioned into several reproducible temporal modules, including winter-induced, summer-induced, autumn-induced, and spring-biased programs.

To further define the biological functions represented by these seasonal modules, we selected Clusters 2, 3, 5, and 6 for GO enrichment analysis. Cluster 2, which was strongly induced in winter, was enriched mainly for RNA- and nucleic acid-related functions (**Figure 3B**). In the Biological Process category, the most significantly enriched terms included RNA modification, nucleic acid phosphodiester bond hydrolysis, and broader metabolic processes involving nucleic acids, heterocyclic compounds, and nitrogen-containing compounds. In the Cellular Component category, enriched terms were centered on the nucleolus and intracellular organelle-associated compartments. In the Molecular Function category, the dominant terms included nuclease activity, endonuclease activity, nucleic acid binding, and RNA binding. These findings suggest that Cluster 2 represents a winter-elevated transcriptional module associated with RNA processing and nuclear functions.

A related but temporally distinct functional profile was observed for Cluster 3, which peaked in summer and declined sharply in autumn (**Figure 3B**). This cluster was also enriched for RNA metabolism and nuclear regulatory functions, including nucleic acid metabolic process, RNA modification, and broader macromolecule metabolic processes. Consistent with these biological processes, the enriched cellular components included the nuclear lumen, nucleoplasm, nucleus, and nuclear protein-containing complexes, whereas the enriched molecular functions were dominated by endonuclease activity, nucleic acid binding, RNA binding, and related binding categories. Thus, although Clusters 2 and 3 shared broadly similar functional annotations, they differed clearly in seasonal timing, suggesting that RNA-associated regulatory programs are activated at different phases of the annual cycle.

In contrast, Clusters 5 and 6 represented functionally distinct seasonal programs (**Figure 3B**). Cluster 5, which showed the highest expression in summer followed by a gradual decline toward winter, was enriched mainly for developmental and regulatory processes. In the Molecular Function category, this cluster was enriched for linoleate 13S-lipoxygenase activity and oxidoreductase activity, with additional representation of transcription factor and transcription regulator functions. By contrast, Cluster 6, which was activated in summer and autumn but strongly repressed in winter, was highly enriched for cell wall biosynthetic and structural pathways. The dominant Biological Process terms included plant-type secondary cell wall biogenesis, cell wall biogenesis, cell wall organization or biogenesis, plant-type cell wall biogenesis, and xylan metabolic process.

### Transcriptional differences among oil-yield types

**Figure 4:**
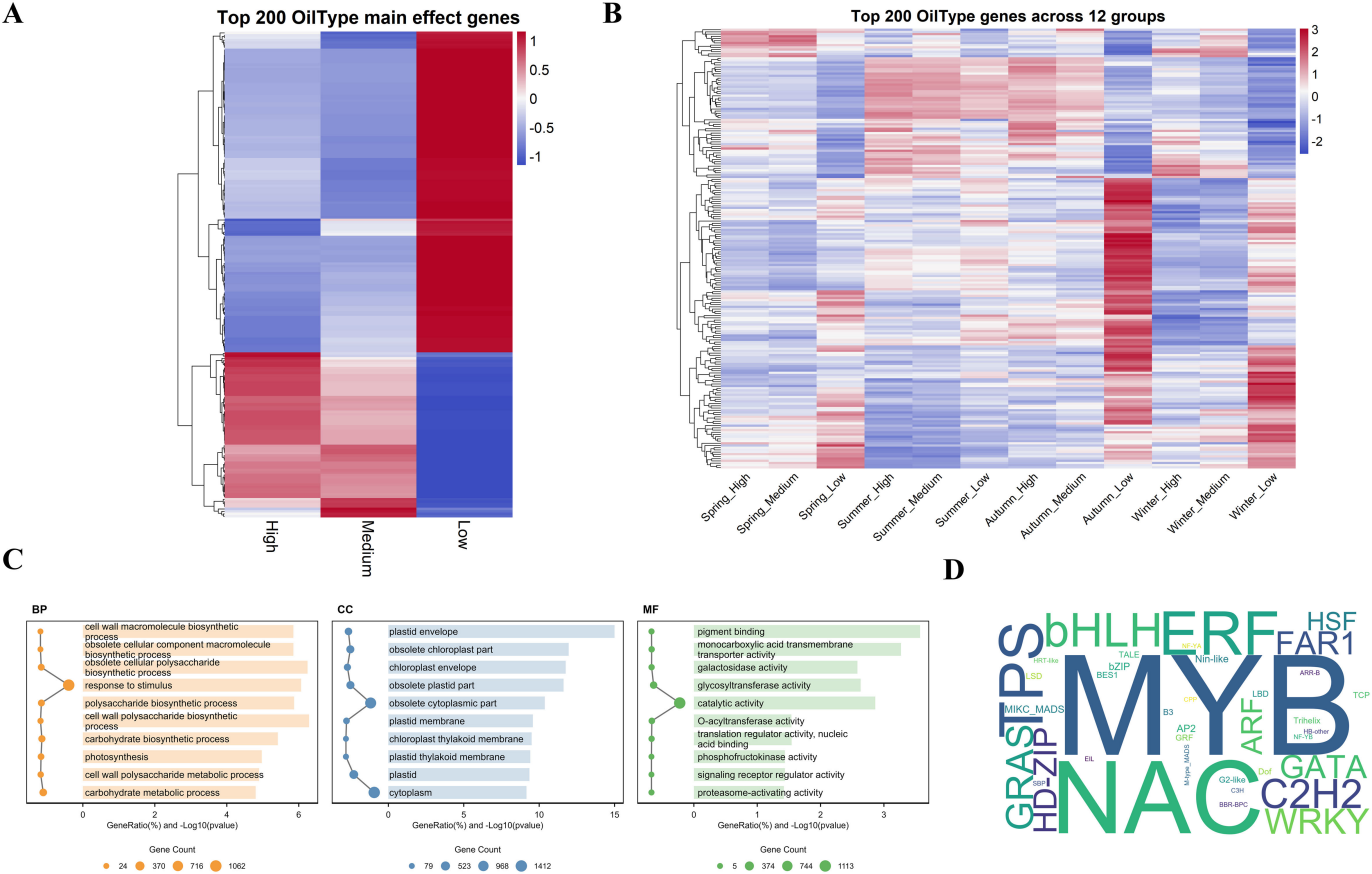
Expression patterns, functional enrichment, and transcription factor composition of oil-type-responsive genes in *Camphora longepaniculata*. **(A)** Heatmap of the top 200 OilType-responsive genes across high-, medium-, and low-oil classes. **(B)** Heatmap showing the expression profiles of the same genes across all 12 season-by-oil-type combinations. The color scale represents relative standardized expression levels. **(C)** Gene Ontology (GO) enrichment analysis of genes identified from the OilType main-effect test. Bubble size reflects gene number, and bubble color indicates enrichment significance. **(D)** Word cloud showing the distribution of transcription factor families among significant oil-type-responsive genes identified from the multifactor DESeq2 main-effect analysis. Term size is proportional to the number of differentially expressed transcription factor genes in each family.

To characterize transcriptional features associated with oil-yield class independently of seasonal background, we examined the main effect of OilType in a multifactor DESeq2 model in which oil type was tested while controlling for season. Significant oil-type-responsive genes identified by the likelihood ratio test were then visualized in heatmaps based on the top 200 genes ranked by adjusted *P* value (**Figure 4A**). When expression was summarized across the three oil-yield classes, these genes separated into two major reciprocal expression groups. One large group showed relatively high expression in the low-oil line but lower expression in the high- and medium-oil lines, whereas the other showed the opposite pattern, with higher expression in the high- and medium-oil lines and lower expression in the low-oil line. In this comparison, the medium-oil line more closely resembled the high-oil line than the low-oil line, indicating that the main transcriptional contrast associated with oil type was driven largely by the divergence of the low-oil material from the other two oil-yield classes.

This overall pattern remained evident when the same gene set was examined across all season-by-oil-type combinations (**Figure 4B**). Although seasonal variation introduced additional differences in expression amplitude, many genes maintained broadly consistent opposite trends between the low-oil line and the high-/medium-oil lines across multiple seasonal backgrounds. In particular, several gene clusters showed stronger signals in low-oil samples collected in autumn or winter, whereas other groups displayed relatively stable enrichment in the high- and medium-oil lines throughout the annual cycle. These results indicate that the OilType main effect reflects a robust transcriptional signature associated with oil-yield class, although the strength of this signature is modulated by season.

To further define the biological functions represented by these oil-type-associated genes, we performed GO enrichment analysis on the significant genes identified from the OilType main-effect test (**Figure 4C**). In the Biological Process category, enriched terms were dominated by cell wall macromolecule biosynthetic process, polysaccharide biosynthetic process, cell wall polysaccharide biosynthetic process, carbohydrate derivative biosynthetic process, carbohydrate metabolic process, photosynthesis, and response to stimulus. In the Cellular Component category, the most prominent terms were associated with the plastid envelope, chloroplast envelope, plastid membrane, chloroplast thylakoid membrane, plastid thylakoid membrane, and plastid, indicating strong enrichment of genes functioning in plastid- and chloroplast-related compartments. In the Molecular Function category, enriched terms included pigment binding, glycosyltransferase activity, O-acyltransferase activity, galactosidase activity, monocarboxylic acid transmembrane transporter activity, transition metal ion transmembrane transporter activity, and phosphofructokinase activity. Together, these enrichment profiles indicate that transcriptional differences associated with oil-yield type are linked not only to regulatory variation, but also to plastid-associated metabolism, carbohydrate and cell wall biosynthesis, and transport-related activities.

Because transcriptional regulation may contribute to these stable differences among oil-yield classes, the significant OilType-responsive differentially expressed genes were screened for putative transcription factors. These transcription factors were therefore identified from significant differentially expressed genes associated with oil-yield class. This analysis identified 158 predicted transcription factor genes belonging to 38 families (**Figure 4D**). The largest family was MYB (n = 27), followed by NAC (n = 12), and ERF, TPS, and bHLH (each n = 11). Other relatively abundant families included C2H2 (n = 8), GRAS (n = 7), WRKY (n = 6), and FAR1, GATA, and HD-ZIP (each n = 5), with smaller contributions from ARF, HSF, AP2, bZIP, MIKC-MADS, Nin-like, and several other families (**Supplementary Table 3**). The prominence of MYB, NAC, ERF, bHLH, WRKY, and related regulators suggests that the oil-yield-associated expression program may be associated with broad transcriptional control involving multiple regulatory modules rather than a single dominant pathway.

### A co-expression module associated with oil yield

**Figure 5:**
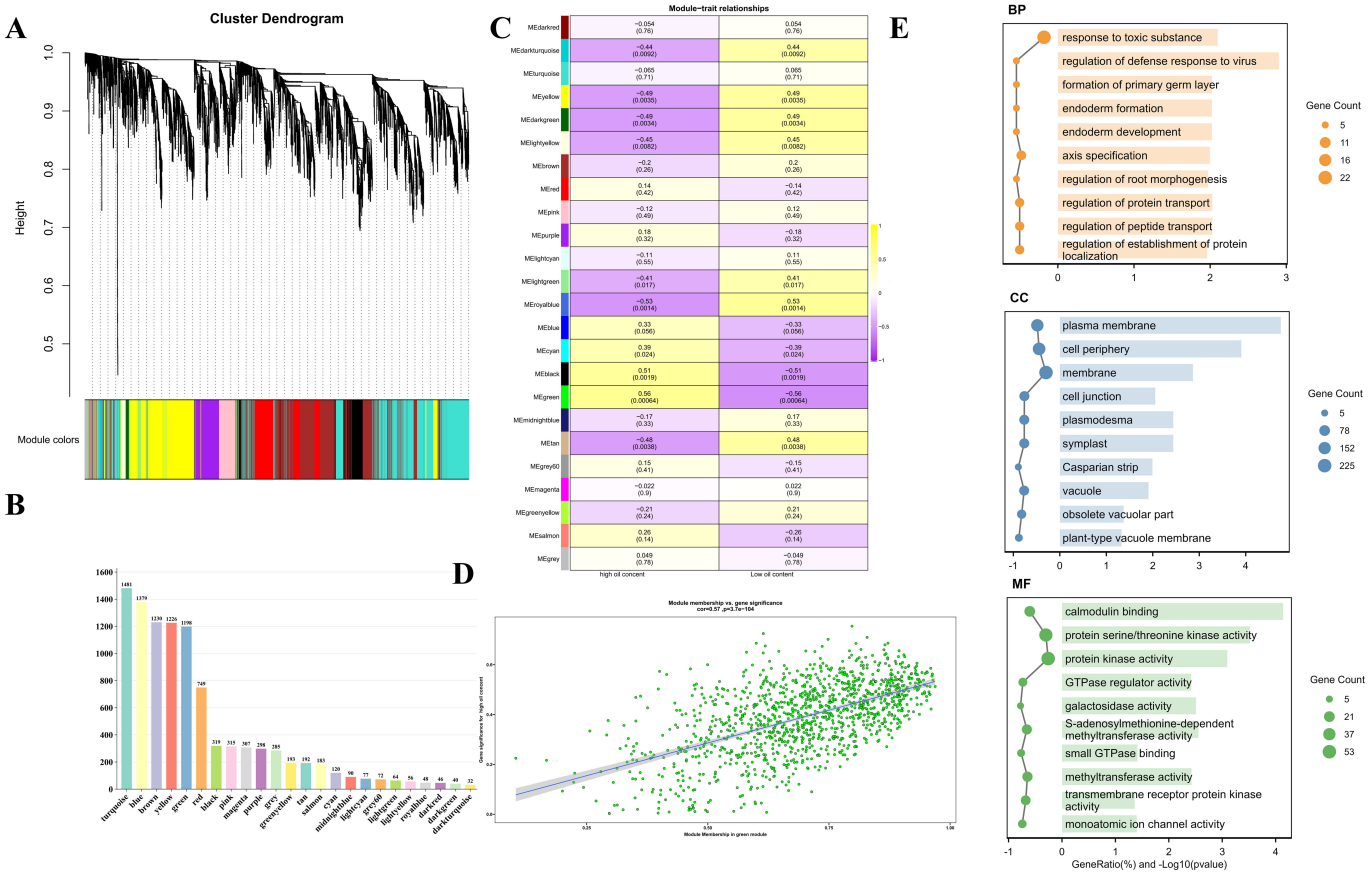
Weighted gene co-expression network analysis of transcriptome data from *Camphora longepaniculata*. **(A)** Hierarchical clustering dendrogram of genes generated for network construction, with module colors shown below the dendrogram. **(B)** Bar plot showing the number of genes assigned to each co-expression module. **(C)** Module–trait correlation heatmap; values indicate correlation coefficients and *P* values. **(D)** Scatter plot showing the relationship between module membership and gene significance for the selected trait-associated module. **(E)** GO enrichment analysis of genes in the selected module. Bubble size indicates gene count, and bubble color represents enrichment significance.

To identify coordinated transcriptional programs associated with oil yield, we performed WGCNA on the transcriptome dataset. The expressed genes were grouped into 23 co-expression modules, in addition to the gray module containing unassigned genes, with module size ranging from 32 to 1,481 genes (**Figures 5A, B**). Among these, the green module, which contained 1,198 genes, showed one of the strongest associations with oil-yield type. This module was positively correlated with high oil content and negatively correlated with low oil content (**Figure 5C**). Module membership within the green module was also positively associated with gene significance, indicating that genes with higher intramodular connectivity tended to show stronger associations with the oil-content trait (**Figure 5D**). Together, these results identify the green module as a trait-relevant co-expression unit correlated with the high-oil phenotype.

To define the biological functions represented in this module, we performed GO enrichment analysis on the green-module genes (**Figure 5E**). In the Biological Process category, enriched terms included response to toxic substance, regulation of defense response to virus, formation of primary germ layer, endoderm formation, axis specification, regulation of root morphogenesis, and regulation of peptide transport. Because the samples analyzed in this study were leaf tissues, these development-related terms should be interpreted cautiously and may reflect broad GO annotations, functional pleiotropy of homologous genes, or annotation transfer limitations rather than direct embryonic developmental processes in leaves. In the Cellular Component category, the enriched terms were mainly associated with the plasma membrane, cell periphery, membrane, cell junction, plasmodesma, symplast, Casparian strip, and vacuole-related structures, indicating a strong membrane-associated signature. In the Molecular Function category, the dominant terms included calmodulin binding, protein serine/threonine kinase activity, protein kinase activity, GTPase regulator activity, galactosidase activity, methyltransferase activity, and monatomic ion channel activity. Taken together, these enrichments suggest that the green module is enriched for genes involved in signaling, membrane-associated processes, and regulatory metabolism.

**Figure 6:**
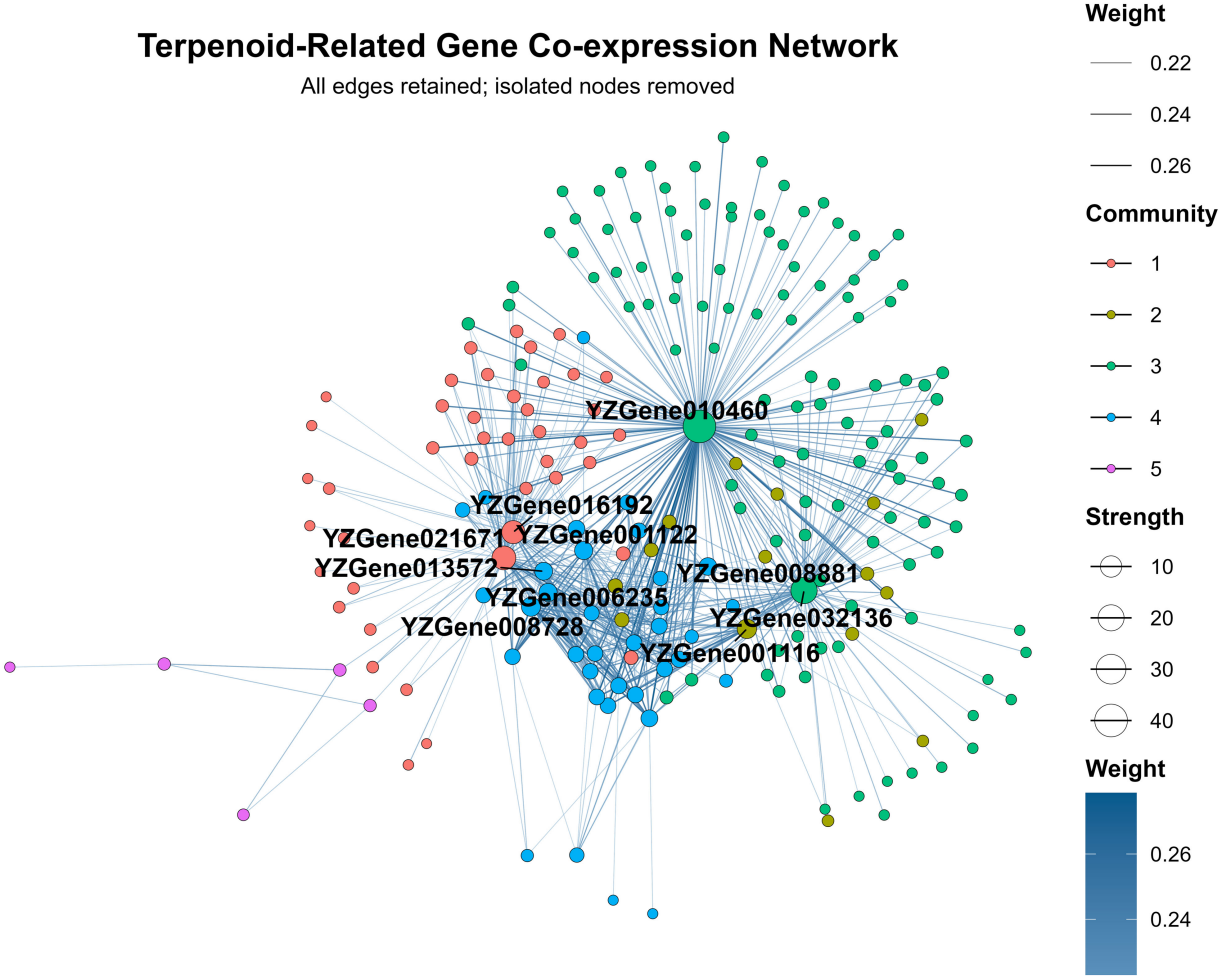
Co-expression network of genes in the selected module. Node size represents connectivity strength, edge width indicates connection weight, and node colors indicate network communities.

**Figure 7:**
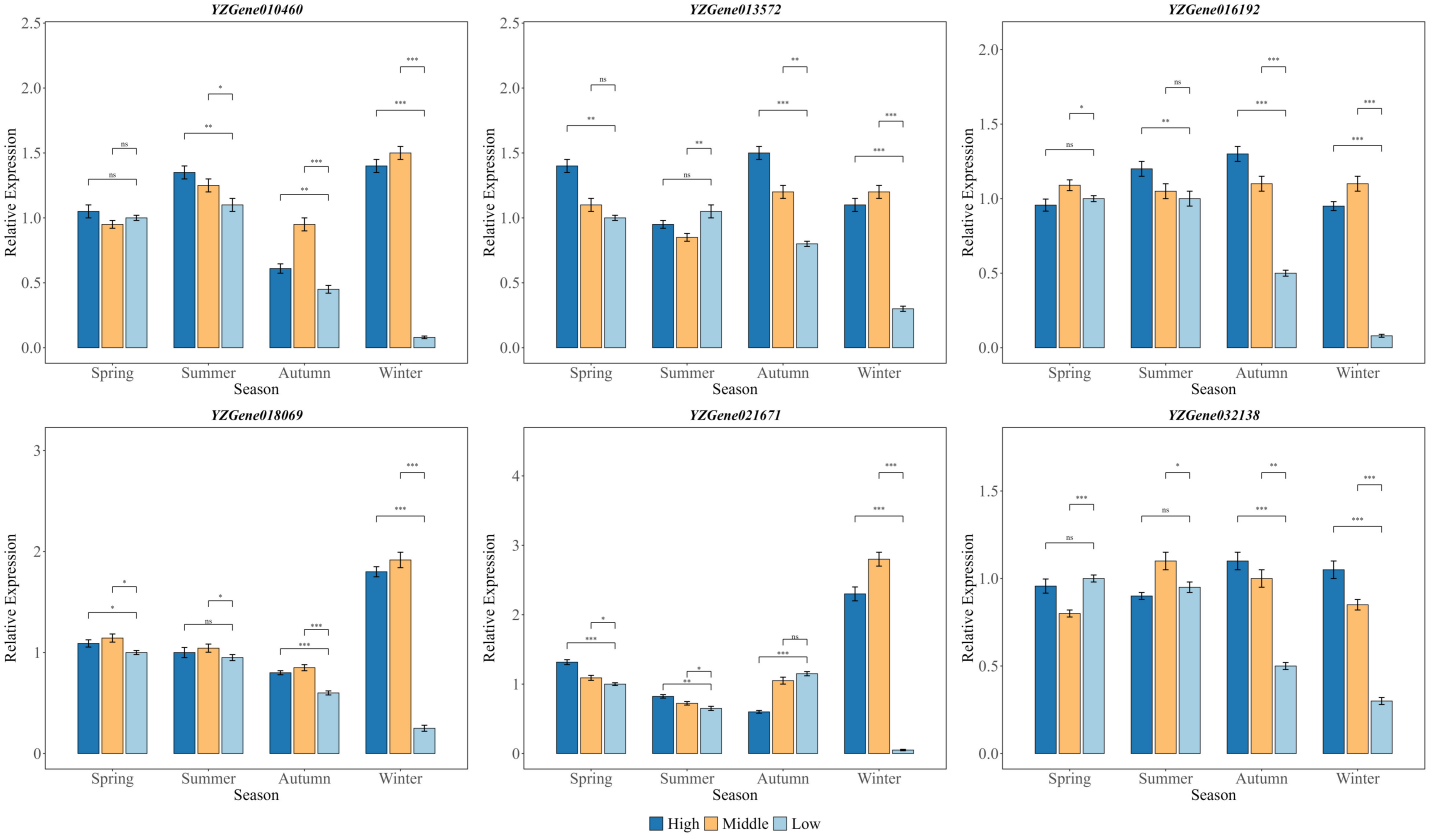
qRT-PCR validation of representative candidate genes in *Camphora longepaniculata*. Relative expression levels of six selected genes were measured by qRT-PCR in high-, medium-, and low-oil lines across spring, summer, autumn, and winter. Expression levels were normalized using Actin as the internal reference gene. Error bars indicate the standard deviation of biological replicates. Asterisks indicate significant differences among groups.

We next examined the intramodular network structure of the green module to identify highly connected candidate genes (**Figure 6**). This analysis highlighted a set of prominent hub-like candidate genes, including ABCG/PDR transporters (*YZGene002917* and *YZGene010460*), SAM-dependent O-methyltransferases (*YZGene008881*, *YZGene020108*, and *YZGene021671*), several cytochrome P450 genes (*YZGene008728*, *YZGene008729*, *YZGene014237*, and *YZGene016192*), aldo-keto reductases (*YZGene001116* and *YZGene001122*), UDP-glycosyltransferases (*YZGene003876*, *YZGene003880*, and *YZGene018069*), a terpene synthase (*YZGene032138*), a short-chain dehydrogenase/reductase (*YZGene037836*), glycerol-3-phosphate dehydrogenase (*YZGene032136*), a squalene monooxygenase-like gene (*YZGene013572*), and a zeaxanthin epoxidase-like gene (*YZGene006235*). Notably, many of these genes encode enzymes or transport-related proteins that are putatively associated with secondary metabolism, redox processes, and metabolite modification. Based on these results, six representative hub genes were selected for qPCR validation (**Figure 7**). These genes were selected to represent highly connected candidates in the green module and different functional annotations. Their expression profiles were generally consistent with the transcriptome data, supporting the reliability of the RNA-seq expression patterns. These results indicate that the selected genes are representative candidates associated with the high-oil phenotype, although their functional roles require further experimental validation.

### Expression patterns of terpene-biosynthetic pathway genes across oil-yield types

**Figure 8:**
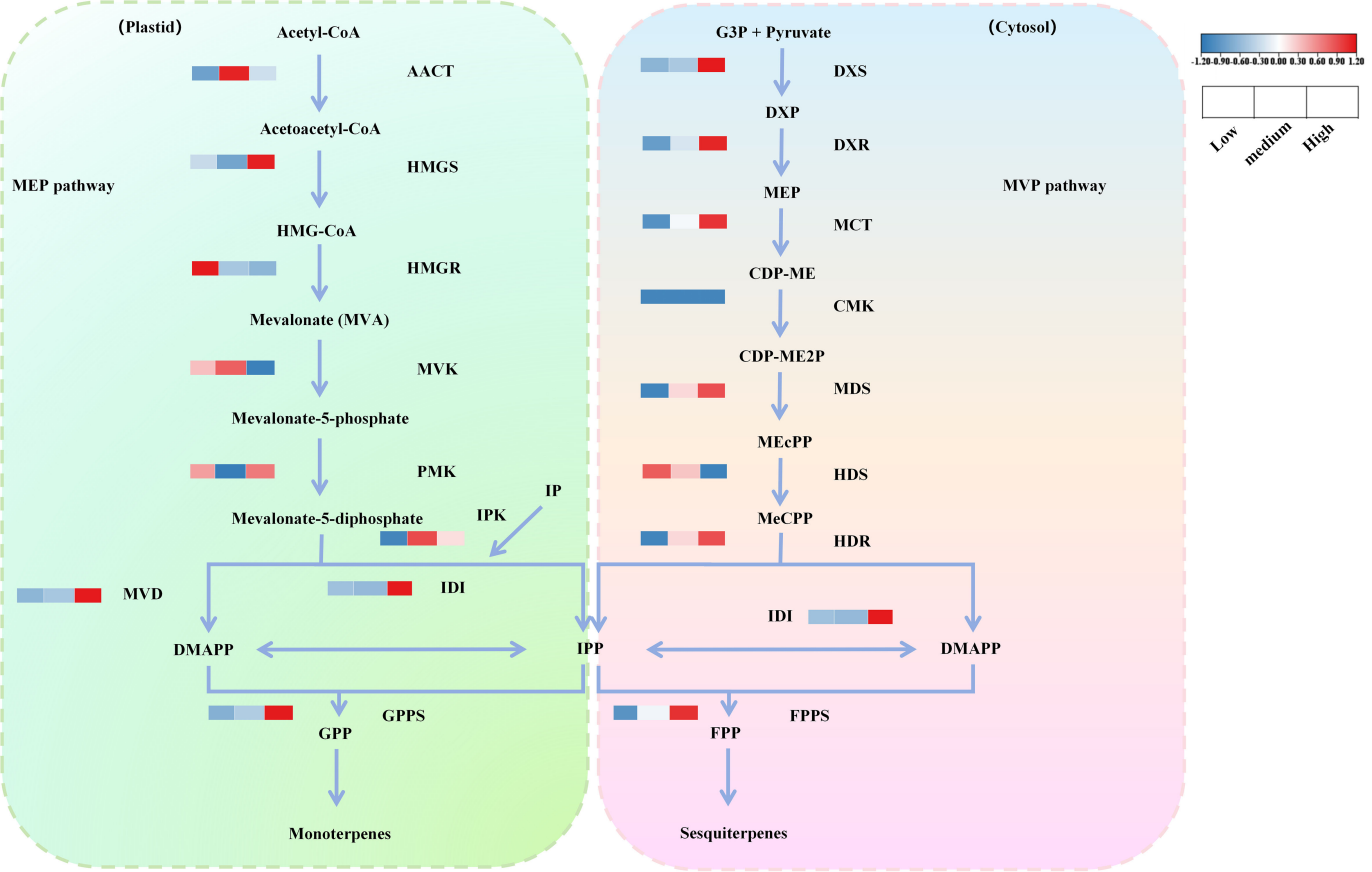
Expression profiles of genes involved in terpene precursor biosynthesis pathways in *Camphora longepaniculata*. Schematic representation of genes assigned to the mevalonate (MVA) and methylerythritol phosphate (MEP) pathways. Colored boxes next to each enzyme indicate relative expression levels in the low-, medium- and high- oil lines, as shown by the color scale.

To examine whether oil-yield classes were associated with transcriptional differences in terpene precursor-related pathways, the expression patterns of genes assigned to the two main isoprenoid precursor-producing pathways, the mevalonate (MVA) pathway and the methylerythritol phosphate (MEP) pathway, were compared across the low-, medium-, and high-oil lines. Overall, the pathway map showed that a substantial proportion of terpene-biosynthetic genes were more highly expressed in the high-oil line, although the extent and direction of change varied across individual enzymatic steps (**Figure 8**).

Within the MVA branch, several genes in the downstream part of the pathway showed elevated expression in the high-oil line, including *HMGS*, *MVD*, *IDI*, and *GPPS.* This pattern suggests a possible association with *DMAPP/IPP* production and *GPP* formation in the high-oil material, although metabolic flux was not directly measured in this study. By contrast, *HMGR* was more highly expressed in the low-oil line, whereas *AACT* and *MVK* showed stronger expression in the medium-oil line. *PMK* showed a less consistent pattern, with relatively higher expression in the low- and high-oil lines than in the medium-oil line. These results indicate that transcriptional changes in the MVA pathway were not uniform across all steps, but were more evident in several downstream genes related to precursor supply.

A similar pattern was observed in the MEP branch. Multiple genes, including *DXS*, *DXR*, *MCT*, *MDS*, *HDR*, *IDI*, and *FPPS*, showed higher expression in the high-oil line than in the low-oil line. This expression pattern is consistent with a possible increased transcriptional activity in the plastidial isoprenoid pathway and in steps contributing to *IPP/DMAPP* interconversion and *FPP* formation. In contrast, *HDS* showed the opposite trend, with relatively stronger expression in the low-oil line. As in the MVA branch, therefore, most but not all steps in the MEP pathway were positively associated with oil yield.

Taken together, this pathway-level comparison indicates that the high-oil line generally showed elevated expression of multiple genes annotated in terpene precursor formation, especially those associated with the generation and downstream use of I*PP/DMAPP*. At the same time, several enzymes showed non-parallel patterns across oil-yield classes, suggesting that variation in oil yield is associated with selective rather than globally uniform transcriptional regulation of terpene biosynthesis. Overall, this expression pattern is consistent with a possible higher precursor-production capacity in the high-oil material and provides pathway-level candidate genes associated with transcriptomic differences observed among oil-yield types.

## Discussion

In this study, RNA-seq was used to examine seasonal transcriptomic variation among high-, medium-, and low-oil-yielding *Camphora longepaniculata* lines and to identify expression features associated with oil-yield classes. The sequencing data were of high quality, with consistent Q20, Q30, GC content, error rate, and mapping rate values across samples, supporting their suitability for comparative transcriptomic analysis. Correlation analysis, PCA, and hierarchical clustering consistently showed that samples were separated primarily by season rather than by oil-yield type. This indicates that season is the dominant factor shaping the global transcriptomic landscape of *C. longepaniculata* leaves. By contrast, oil-yield-specific effects represented a more stable but secondary component of transcriptional variation. The high- and medium-oil lines were transcriptionally more similar to each other than to the low-oil line, suggesting that the main oil-yield-associated difference was driven largely by the divergence of the low-oil material rather than by a gradual expression gradient across oil-yield levels. Thus, the seasonal effect mainly reflected broad annual transcriptome reprogramming, whereas the oil-yield effect captured relatively stable differences among oil-yield classes.

The strong seasonal separation observed here is consistent with previous transcriptomic studies in perennial plants. Seasonal transcriptome analyses in tea leaves and Norway spruce needles have shown that annual environmental changes can substantially reorganize gene expression programs related to photosynthesis, carbohydrate metabolism, stress responses, and growth regulation (Kumar et al., 2016; Bag et al., 2021). In line with this, season-responsive genes in the present study were grouped into seven temporal expression clusters, indicating that seasonal regulation in *C. longepaniculata* consists of multiple coordinated transcriptional programs rather than a single dominant pattern. These seasonal programs may also have ecological relevance. Because the distribution and physiology of *C. longepaniculata* are sensitive to climatic factors such as temperature and precipitation (Zhu et al., 2025), seasonal transcriptome reprogramming may represent an important molecular layer through which this species adjusts to annual environmental variation. The relatively dispersed distribution of autumn samples may reflect a transitional physiological state between active growth and winter adjustment, although this remains to be tested experimentally.

Differential expression analysis showed that transcriptional differences associated with oil-yield classes are strongly influenced by season. Seasonal comparisons within each oil-yield type revealed strong annual expression changes, indicating that seasonal reprogramming was a major source of transcriptomic variation. In contrast, comparisons among oil-yield types within the same season showed that oil-yield-associated differences were most evident in autumn and winter, especially in comparisons involving the low-oil line. The high-versus-medium comparison produced far fewer DEGs, indicating that the high- and medium-oil lines shared more similar expression features. These results suggest that seasonal effects reflect broad temporal transcriptome shifts, whereas oil-yield-associated effects are most strongly expressed as the divergence of the low-oil line from the high- and medium-oil lines under specific seasonal backgrounds. Previous studies in *Cinnamomum* species have linked variation in essential oil accumulation to differential expression of genes involved in terpenoid biosynthesis and secondary metabolism (Yan et al., 2017; Zhao et al., 2022). The present results extend these transcriptome-level findings by showing that such differences are not constant throughout the year but are modulated by seasonal context.

Seasonal main-effect analysis identified several biologically distinct expression programs. Cluster 2 was strongly induced in winter and enriched in RNA modification, nucleic acid metabolism, nucleolus-associated components, nuclease activity, and RNA binding, suggesting enhanced RNA processing and nuclear regulatory activity under winter conditions. In plants, seasonal acclimation often involves extensive transcriptional and post-transcriptional reprogramming (Zhao et al., 2022), and the observed winter induction of RNA-related functions may reflect increased regulatory adjustment. Clusters 3 and 5 showed summer-biased expression. Cluster 3 was enriched for RNA metabolism and nuclear regulatory functions, whereas Cluster 5 was associated with developmental processes, oxidoreductase activity, lipoxygenase activity, and transcriptional regulation. This suggests that summer is associated with active regulatory metabolism and growth-related processes in *C. longepaniculata* leaves. Cluster 6 was induced in summer and autumn but repressed in winter and was enriched in secondary cell wall biogenesis, cell wall organization, and xylan metabolism, indicating that cell wall formation and structural remodeling are more active during warmer growth periods. This is consistent with previous studies showing that secondary cell wall biosynthesis is closely linked to plant growth and structural development (Zhong and Ye, 2015). Together, these seasonal modules suggest that C. longepaniculata leaves undergo coordinated transcriptional adjustments related to regulatory control, growth, stress response, and structural remodeling across the annual cycle. Such transcriptome plasticity may contribute to ecological adjustment under seasonal climatic variation.

The OilType main-effect analysis identified a stable transcriptional signature associated with oil-yield class after accounting for seasonal effects. The significant genes formed two reciprocal expression groups, one more highly expressed in the low-oil line and the other in the high- and medium-oil lines. This result indicates that oil-yield-specific transcriptional differences were not simply a consequence of seasonal variation, but represented a relatively stable expression signature separating the low-oil material from the high- and medium-oil materials. Functional enrichment analysis showed that these genes are associated with cell wall and polysaccharide biosynthesis, carbohydrate metabolism, photosynthesis, plastid and chloroplast components, pigment binding, glycosyltransferase and O-acyltransferase activities, transport, and phosphofructokinase activity. These results suggest that oil-yield differences in *C. longepaniculata* are linked not only to terpenoid biosynthesis but also to broader metabolic processes, including plastid function, photosynthetic carbon supply, carbohydrate metabolism, cell wall biosynthesis, and metabolite transport. This is consistent with the fact that plant terpenoids are synthesized from IPP and DMAPP, which are produced via the cytosolic mevalonate pathway and the plastidial methylerythritol phosphate pathway (Vranová et al., 2013). Monoterpene biosynthesis is generally associated with plastidial precursor supply, whereas sesquiterpene biosynthesis is mainly linked to the cytosolic MVA pathway, although exchange between compartments can occur (Nagegowda, 2010). The enrichment of plastid- and chloroplast-related functions among OilType-responsive genes therefore supports a role for plastidial metabolism in determining essential oil accumulation.

A total of 158 putative transcription factor genes from 38 families were identified among the OilType-responsive genes, with MYB, NAC, ERF, TPS, bHLH, C2H2, GRAS, and WRKY families being relatively abundant. Previous studies have shown that MYB, bHLH, ERF, and WRKY transcription factors are involved in regulating plant secondary metabolism and stress responses (Nakano et al., 2006; Rushton et al., 2010). The presence of multiple transcription factor families suggests that oil-yield variation may be associated with a broad regulatory network rather than a single key regulator.

WGCNA identified a green module that was positively associated with high oil yield. The positive relationship between module membership and gene significance indicates that highly connected genes in this module tend to be more strongly associated with the oil-yield trait. Functional enrichment analysis showed that this module is related to membrane-associated structures, signal transduction, cellular communication, and regulatory metabolism, including plasma membrane, plasmodesmata, vacuole-related structures, kinase activity, GTPase regulation, methyltransferase activity, and ion channel activity. Some enriched Biological Process terms in the green module, including formation of primary germ layer, endoderm formation, and axis specification, are not readily interpretable as literal developmental processes in mature leaf tissue. These terms may arise because GO annotations are often transferred from homologous genes characterized in other species or developmental contexts, and because some genes involved in signaling or cellular regulation can be annotated to broad developmental categories. Therefore, these development-related terms were treated cautiously and were not used them as primary evidence for the biological interpretation of oil-yield variation. Several hub-like genes in the green module encode ABCG/PDR transporters, SAM-dependent O-methyltransferases, cytochrome P450s, aldo-keto reductases, UDP-glycosyltransferases, terpene synthases, short-chain dehydrogenase/reductases, glycerol-3-phosphate dehydrogenase, and enzymes related to squalene and carotenoid metabolism. Based on their annotations, these genes may be associated with secondary metabolism, redox reactions, metabolite modification, and transport. Because plant volatile compounds often undergo enzymatic modification and require transport for accumulation and storage (Dudareva et al., 2013), this module may represent a coordinated metabolic network associated with the high-oil phenotype. However, this inference is based on correlation analysis, and the functional roles of these hub genes require experimental validation.

Analysis of terpenoid biosynthetic pathway genes provided additional support for oil-yield-associated transcriptional differences. In the MVA pathway, several downstream genes, including HMGS, MVD, IDI, and GPPS, were more highly expressed in the high-oil line, suggesting enhanced capacity for IPP/DMAPP production and GPP formation. In the MEP pathway, DXS, DXR, MCT, MDS, HDR, IDI, and FPPS also showed higher expression in the high-oil line, consistent with increased plastidial isoprenoid precursor supply. Because pathway flux, enzyme activities, and terpene metabolite levels were not directly measured in the same seasonal samples, these interpretations should be considered transcriptome-based candidate associations rather than confirmed metabolic mechanisms. Notably, DXS and DXR are known to influence flux through the MEP pathway (Rodrıguez-Concepción and Boronat, 2002), supporting this interpretation. Similar relationships between terpenoid pathway gene expression and volatile oil accumulation have been reported in other aromatic plants, including lavender and *Amomum villosum* (Mendoza-Poudereux et al., 2017; Li et al., 2024). The present results are consistent with these findings, although the expression pattern in *C. longepaniculata* is not uniform across all pathway steps, indicating a more complex association between pathway gene expression and oil-yield variation. Importantly, not all terpenoid pathway genes were more highly expressed in the high-oil line. For example, HMGR showed higher expression in the low-oil line, AACT and MVK were more highly expressed in the medium-oil line, and HDS showed stronger expression in the low-oil line. These patterns suggest that oil-yield variation is not due to uniform upregulation of the MVA or MEP pathways but rather reflects selective regulation at specific steps, together with differences in precursor allocation, pathway interactions, compartmental exchange, and downstream modification or transport. This is consistent with the general view that plant terpenoid metabolism is regulated by multiple factors, including pathway flux, substrate availability, enzyme specificity, compartmentalization, and environmental context (Tholl, 2006).

Overall, this study shows that oil-yield classes in *C. longepaniculata* leaves are associated with two distinguishable layers of transcriptional variation. The first layer is the seasonal effect, which was the primary driver of global gene expression and reflected broad transcriptome reprogramming across the annual cycle. The second layer is the oil-yield-associated effect, which was more stable and was reflected mainly in the divergence of the low-oil line from the high- and medium-oil lines after accounting for seasonal variation. The transcriptional features associated with high oil yield were related to terpene precursor biosynthesis, plastid metabolism, photosynthesis, carbohydrate metabolism, cell wall biosynthesis, membrane-associated signaling, transcriptional regulation, and metabolite modification and transport. These findings provide a set of candidate genes and regulatory modules for further study. It should be noted that the current study is based mainly on transcriptomic association analysis. Therefore, the relationships between transcript abundance, hub genes, transcription factors, and oil accumulation should be interpreted as correlative rather than causal. Although several candidate genes and co-expression modules were associated with oil-yield class, their functional roles in essential-oil biosynthesis or accumulation were not directly tested in this study. Future work should combine seasonal oil-content measurements, targeted metabolite profiling, enzyme activity assays, and functional validation through gene overexpression, silencing, or genome editing to confirm the roles of these candidates in oil accumulation.

## Data Availability

The datasets presented in this study can be found in online repositories. The names of the repository/repositories and accession number(s) can be found below: https://www.ncbi.nlm.nih.gov/, PRJNA1458560.
